# The Causes of Extra Uterine Pregnancy

**Published:** 1901-08-10

**Authors:** 


### THE CAUSES OF EXTRA UTERINE PREGNANCY.

In a lecture on the early symptoms of ectopic pregnancy Dr. Neil Macphatter[1] gives a good sketch of the modern view of the manner in which this accident arises, and the conditions necessary for its production. He says that the ovular theory of menstruation that was for so long accepted by the profession has greatly hindered progress in the elucidation of this and kindred diseases. Ovulation and menstruation should be considered as absolutely independent of each other. Ovulation is a constant function of the ovaries, and is in progress long before and after menstruation begins and ends. Menstruation is an occasional function of the uterus, and is limited to the child-bearing period of the life of a woman. Our text-books, he says, are teeming with erroneous teachings on these subjects, the majority of authorities maintaining that after an ovum is grasped by the fimbriated extremity of the Fallopian tube it remains quiescent in the lumen of the tube until such time as a spermatozoon comes along and there impregnates it. He holds, however, that it is probable that whenever an ovum matures sufficiently and is ready to leave the ovary, it is grasped by the fimbriated extremity and is sent on its way down the tube to the uterine cavity, irrespective of the presence of spermatozoa or the condition of the mucous membrane of the uterus. Should such an ovum meet a healthy spermatozoon in the uterine cavity it would become vitalised and go on to develop if the mucous membrane of the uterus were in a suitable condition. Otherwise the ovum is cast off and passes away in solution. The great majority of ova pass away in this fashion. It is the coincidence of a living ovum, a living spermatozoon, and a surface to which the impregnated ovum is capable of becoming attached, that is necessary to ensure the conception. The Fallopian tubes and the uterine cavity are lined with epithelium, the particular variety of which is peculiar and suggestive. That lining the tube and the upper part of the uterine cavity is ciliated. In the lower part of the uterus it is stratified. It is a mistaken idea that at each menstrual period the mucous membrane is cast off. It has been established beyond dispute that it is only the epithelial covering of the uterus and that covering the mouths of the utricular glands that is shed. Occasionally, as in cases of membraneous dysmenorrhœa, the entire mucous membrane may come away; but normally only the epithelial lining is exfoliated. The subjacent mucous membrane becomes swollen, engorged, and puffy, "and ready for a fertilised ovum to nestle in its velvety surface." The epithelial lining of the tubes takes no active part in the process of menstruation. The condition of the epithelium of the uterus plays a very important part in the etiology of normal pregnancy. Epithelium has no blood-vessels and receives its nourishment only from the lymph which exudes from the subjacent blood-vessels. It is necessary then that the epithelial covering should be removed before an ovum can become attached. In the uterus this is accomplished once a month by a process of shedding, which occurs at the menstrual period. It may be accepted then that an ovum cannot become attached to an epithelial surface, and so long as the tubes remain in a healthy normal condition it will be impossible for tubal pregnancy to take place. It becomes necessary to destroy its ciliated epithelium before such a pregnancy can occur, and thus it is that a previous salpingitis, the result frequently of a latent gonorrhœa, a miscarriage, or an abortion, are frequent precursors of a tubal pregnancy. When the ovum becomes attached near the fringes of the tube the rupture is frequently early and into the peritoneal cavity. Occasionally the attachment of the fœtus occurs in that part of the tube that passes through the wall of the uterus. It is then called interstitial and is extremely difficult to diagnose.

[1] The Post-Graduate, July, 1901.

